# Changes in fatty acid composition of *Stenotrophomonas maltophilia* KB2 during co-metabolic degradation of monochlorophenols

**DOI:** 10.1007/s11274-016-2160-y

**Published:** 2016-10-18

**Authors:** Agnieszka Nowak, Izabela Greń, Agnieszka Mrozik

**Affiliations:** Department of Biochemistry, Faculty of Biology and Environmental Protection, University of Silesia, Jagiellońska 28, 40-032 Katowice, Poland

**Keywords:** Co-metabolism, Fatty acid composition, Monochlorophenols, *Stenotrophomonas maltophilia* KB2

## Abstract

The changes in the cellular fatty acid composition of *Stenotrophomonas maltophilia* KB2 during co-metabolic degradation of monochlorophenols in the presence of phenol as well as its adaptive mechanisms to these compounds were studied. It was found that bacteria were capable of degrading 4-chlorophenol (4-CP) completely in the presence of phenol, while 2-chlorophenol (2-CP) and 3-chlorophenol (3-CP) they degraded partially. The analysis of the fatty acid profiles indicated that adaptive mechanisms of bacteria depended on earlier exposure to phenol, which isomer they degraded, and on incubation time. In bacteria unexposed to phenol the permeability and structure of their membranes could be modified through the increase of hydroxylated and cyclopropane fatty acids, and straight-chain and hydroxylated fatty acids under 2-CP, 3-CP and 4-CP exposure, respectively. In the exposed cells, regardless of the isomer they degraded, the most important changes were connected with the increase of the contribution of branched fatty acid on day 4 and the content of hydroxylated fatty acids on day 7. The changes, particularly in the proportion of branched fatty acids, could be a good indicator for assessing the progress of the degradation of monochlorophenols by *S. maltophilia* KB2. In comparison, in phenol-degrading cells the increase of cyclopropane and straight-chain fatty acid content was established. These findings indicated the degradative potential of the tested strain towards the co-metabolic degradation of persistent chlorophenols, and extended the current knowledge about the adaptive mechanisms of these bacteria to such chemicals.

## Introduction

Chlorophenols and their derivatives are ubiquitous contaminants of air, water and soil. They enter the environment through human activities, such as waste incineration, the use of wood preservatives, leather conditioners, solvents, dyes, pesticides and antiseptics, as well as the bleaching of pulp with chlorine (Czaplicka 2004). Due to their high toxicity, strong emissions, persistence in the environment and suspected carcinogenic and mutagenic properties, over recent decades chlorophenols have become quantitatively significant contaminants, listed as priority pollutants by the Agency for Toxic Substances and Disease Registry (ATSDR) and the World Health Organization (WHO).

The fate of chlorophenols in the environment is of great importance and should be constantly monitored. The uncontrolled release of these chemicals with effluents and residues from industrial plants affects the continuous contamination of air, water, soil and sediments, and as a consequence threatens human health (Michałowicz and Duda 2007). This causes a real risk of chronic exposure of living organisms to these compounds, and therefore their disposal and degradation have become a serious challenge. Several physical and chemical methods, including activated carbon adsorption, ion exchange and incineration, have been proposed for treating or recovering chlorophenolic compounds (Hamdaoui and Naffrechoux 2007; Bakhtiar et al. 2011). They are effective but expensive, and can lead to the formation of more harmful intermediates. An alternative to such methods seems to be microbial degradation (Pandey et al. 2009; Mrozik and Piotrowska-Seget 2010). Although biological treatments of chlorophenols with specialized microorganisms are documented in the literature, their use on a large scale is still limited (Balfanz and Rehm 1991; Männistö et al. 2001; Caliz et al. 2011).

The pollution of various environments by a single contaminant is quite rare. More frequently they are contaminated with a mixture of substances with different physicochemical properties and susceptibility to biodegradation (Brack et al. 2003; Antizar-Ladislao et al. 2008; Li et al. 2013). When chlorophenols are present in the mixture and the additional carbon sources are available, their co-metabolic degradation may be possible (Hazen 2010; Vrchotová et al. 2013). The co-substrates can be either the conventional carbon sources supporting cell growth, or the structural analogue compounds inducing the catabolic enzymes (Lee and Lee 2007; Bhatkal et al. 2012).

Among living organisms bacteria have a unique ability to adapt to changing environmental factors, including chemical stress. In response to exposure to harmful compounds, they can modify the structure and fluidity of the cytoplasmic membrane, the first site of contact between cell and chemicals. In the presence of aromatic compounds the changes in saturation degree, length of acyl chains, isomerisation of *cis* to *trans* unsaturated fatty acids, and alterations in the content of cyclopropane, hydroxylated and branched fatty acids in the bacterial membrane are well documented (Duldhardt et al. 2010; Segura et al. 2010; Smułek et al. 2015). The increase in membrane saturation proceeds only in the growing cells as the effect of the *de novo* biosynthesis of saturated fatty acids, and thus it can be a potential marker of harmful effects of chemicals on living cells (Murínová and Dercová 2013). A similar effect, independent of cell growth, is observed when *trans*-unsaturated fatty acids are incorporated into the membrane in a place of *cis*-unsaturated fatty acids. *Cis* to *trans* isomerisation catalysed by *cis*–*trans* isomerase is observed less than 10 min after the contact of cells with the toxic compound, and therefore it is considered a very rapid adaptation mechanism (Heipieper et al. 2003; Bernal et al. 2007). The increase of membrane polarity may be achieved by the increase of hydroxylated acid content. It is generally known that 3-hydroxyl fatty acids are typical intermediates in fatty acid synthesis (López-Lara and Geiger 2010). Conversely, the *S*-2-hydroxylation catalysed by LpxO dioxygenase is post-synthetic modification, usually observed when the fatty acyl group binds to the lipid A molecule (Gibbons et al. 2008). Cyclopropanation of *cis*-unsaturated fatty acids is also a post-synthesis process. Cyclopropane fatty acids are formed by the addition of a methylene group, derived from *S*-adenosyl methionine to the carbon–carbon double bond of unsaturated fatty acids (Segura et al. 2010). It is commonly believed that cyclopropane rings are less reactive than double bonds in their precursors, and the increase of cyclopropane fatty acid abundance has the same effect as the increase in bilayer thickness, and therefore it makes the membrane less permeable (Chang and Cronan 1999). The content of branched fatty acids is also changed in bacteria in the presence of aromatic compounds. The alterations in the ratio of *iso* to *anteiso* branched fatty acids connected with different transition temperatures and stereospecificity of these isomers also affect membrane fluidity (Unell et al. 2007).

The aim of this study was to investigate the composition of the cellular fatty acids of *Stenotrophomonas maltophilia* KB2 during the co-metabolic degradation of monochlorophenols in the presence of phenol as an additional carbon source in batch cultures. It was also interesting to find out if the earlier contact of bacteria with phenol could affect the course of co-metabolic degradation of monochlorophenols and cause significant alterations in fatty acid methyl ester (FAMEs) profiles as compared to the unexposed cells. After a detailed analysis of fatty acid patterns, the adaptive mechanisms of KB2 to the presence of phenol and monochlorophenols have been proposed.

## Materials and methods

### Bacterial strain and culture conditions

The bacterial strain used in this study was *Stenotrophomonas maltophilia* KB2 (VTT E-113197). It is an aerobic, motile, nonfermentative Gram-negative bacterium, isolated from activated sludge of a sewage treatment plant in Bytom-Miechowice (Poland) and described by Guzik et al. (2009) *S. maltophilia* KB2 is known to metabolise broad range of aromatic compounds including phenol, some chloro- and methylphenols, benzoic acids, catechols and others.

In the present study, to assess if the presence of phenol (P) can accelerate the degradation of monochlorophenols, bacteria were grown for 7 days in a mineral salts medium (Mrozik et al. 2007) containing two carbon sources—a proper isomer (2-CP, 3-CP or 4-CP) as the non-growth substrate and P as the growth substrate. Each monochlorophenol was added to the culture medium at a concentration of 130 mg l^−1^. To obtain the molar ratio of 3:1 between the growth and the non-growth substrate, P was added to the medium at a concentration of 280 mg l^−1^. Such ratio was considered as the most appropriate for the co-metabolic degradation of chlorophenols in comparison with the other studied (1:1, 2:1, 3:1, 4:1 and 5:1) (data not shown). When growth or non-growth substrate depletion was confirmed, its subsequent identical dosage was added to the culture. Additionally, the influence of earlier exposure of bacteria to P on their capability to degrade co-metabolically each monochlorophenol was studied. According to Hao et al. (2002), phenol can induce enzymes responsible for the biodegradation of the non-growth substrate. The exposed cells were cultivated in a medium (Mrozik et al. 2007) supplemented with P as a sole carbon source at a concentration of 282 mg l^−1^. However, the cells cultured in the nutrient broth (Sigma-Aldrich) without contact with P were treated as unexposed cells. The initial number of bacteria regardless of the culture conditions was 5 × 10^8^ ml^−1^. All cultures were carried out in 500 ml flasks on a rotary shaker (130 rpm) at 30 °C.

The microbial counts were determined by the dilution plate count technique using nutrient agar (Sigma-Aldrich). The inoculated plates were incubated at 30 °C for 48 h. The number of bacteria was expressed as log CFU ml^−1^. Data are representative of three individual experiments.

### Determination of aromatic compounds concentration

The aromatic compounds (P, 2-CP, 3-CP and 4-CP) were determined by a Merck Hitachi HPLC equipped with an Ascentis^®^ Express C18 HPLC Column (100 × 4.6 mm), Opti-Solw^®^ EXP precolumn and a DAD detector (Merck Hitachi). The mobile phase was a mixture of acetonitrile, methanol and 1 % acetic acid (20:20:60, v/v/v). The flow rate was 1 ml min^−1^. Chemical compounds in the supernatant were identified and quantified by comparing HPLC retention times and UV–visible spectra with those of external standards. The detection wavelength for the detection of P and monochlorophenols was set at 272 nm.

### Catechol 2,3-dioxygenase activity assay

Parallel to the degradation study, the activity of catechol 2,3-dioxygenase [EC 1.13.11.2] in *S. maltophilia* KB2 cells was measured. The preparation of crude extract for enzyme activity assay was performed according to Wojcieszyńska et al. (2013). Catechol 2,3-dioxygenase activity was estimated by the spectrophotometric method (Hegeman 1966) using catechol as a substrate in a reaction mixture. The specific activity of the enzyme was expressed as the number of enzyme units per milligram of protein. The protein concentration in the crude extract was determined by the Bradford method (Bradford 1976) using lysozyme as a standard.

### MIDI-FAME analysis

To determine the composition of the whole-cell derived fatty acids of *S. maltophilia* KB2, FAMEs were directly extracted from phenol-degrading bacteria and control cells (cultivated in nutrient broth) in the late exponential phase of growth (after 6 h) as well as from the batch cultures conducted under co-metabolic conditions on 1, 4 and 7 day. Bacteria were harvested by centrifugation (4500 *g*, 20 min, 4 °C), and next the pellets were washed with 0.9 % NaCl to remove the residue of the culture medium. Fatty acids were extracted according to the procedure by Sasser (1990) and identified using the Microbial Identification System (MIS; Microbial ID Inc., Newark). FAMEs were separated with a gas chromatograph (Hewlett-Packard 6890) equipped with an HP-Ultra 2 capillary column (25 m, 0.22 mm ID) and hydrogen as the carrier gas. FAMEs were detected by a flame ionization detector (FID) and identified using the MIDI Microbial Identification System software (Sherlock TSBA 6.1 method and TSBA6 library; MIDI Inc., Newark, DE, USA).

For the interpretation of the obtained results the identified fatty acids were divided into two groups: saturated (straight-chain, branched, cyclopropane and hydroxylated fatty acids) and unsaturated fatty acids. The subgroup of hydroxylated fatty acids additionally included the hydroxylated, branched fatty acids.

### Data analysis

The results were evaluated by analysis of variance, and statistical analyses were performed on three replicates of data obtained from each treatment. The statistical significance (*p* < 0.05) of differences was treated statistically by two-way ANOVA, considering the effect of substrate and incubation time, and assessed by post hoc comparison of means using the lowest significant differences (LSD) test. The FAMEs profiles were also subjected to principal component analysis (PCA). All data were performed using the Statistica 10.0 PL software package, based on mean values of three replicates.

## Results

### Degradation of phenol and chlorophenols by *S. maltophilia* KB2

**Fig. 1:**
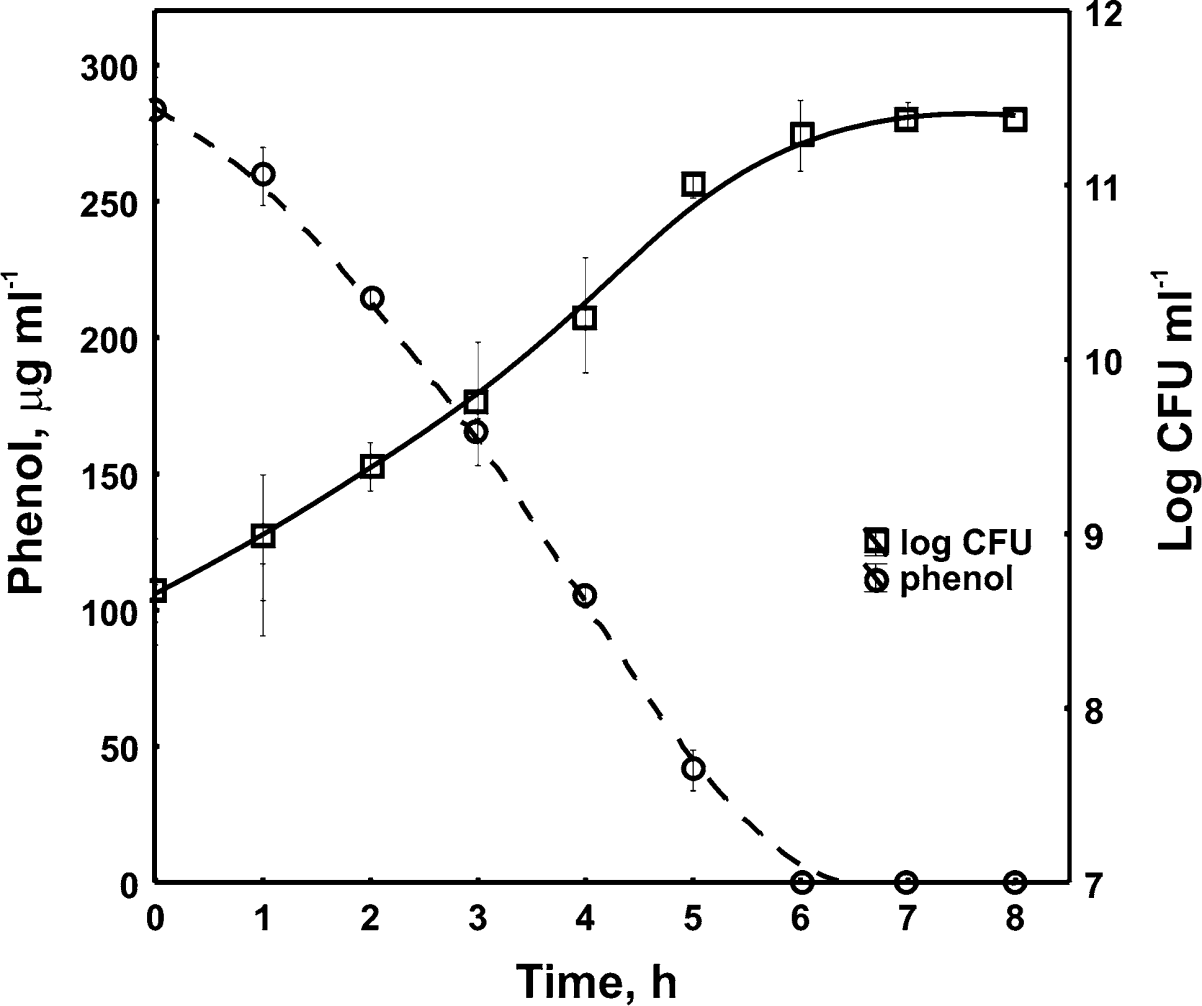
Degradation of phenol (280 mg l^−1^) by *S. maltophilia* KB2 and growth of bacteria. Each *symbol* represents the mean of three replicates

As Guzik et al. (2009) demonstrated, the *S. maltophilia* KB2 strain was not able to degrade monochlorophenols at a concentration of 130 mg l^−1^ as a single carbon source in the batch cultures. Therefore, its ability to degrade 2-CP, 3-CP and 4-CP in the presence of P as a growth substrate was tested. At the beginning, the ability of *S. maltophilia* KB2 to degrade phenol (282 mg l^−1^) was experimentally confirmed. The complete breakdown of P proceeded within 6 h and it was accompanied by a significant increase in the number of cells (Fig. 1).

**Fig. 2:**
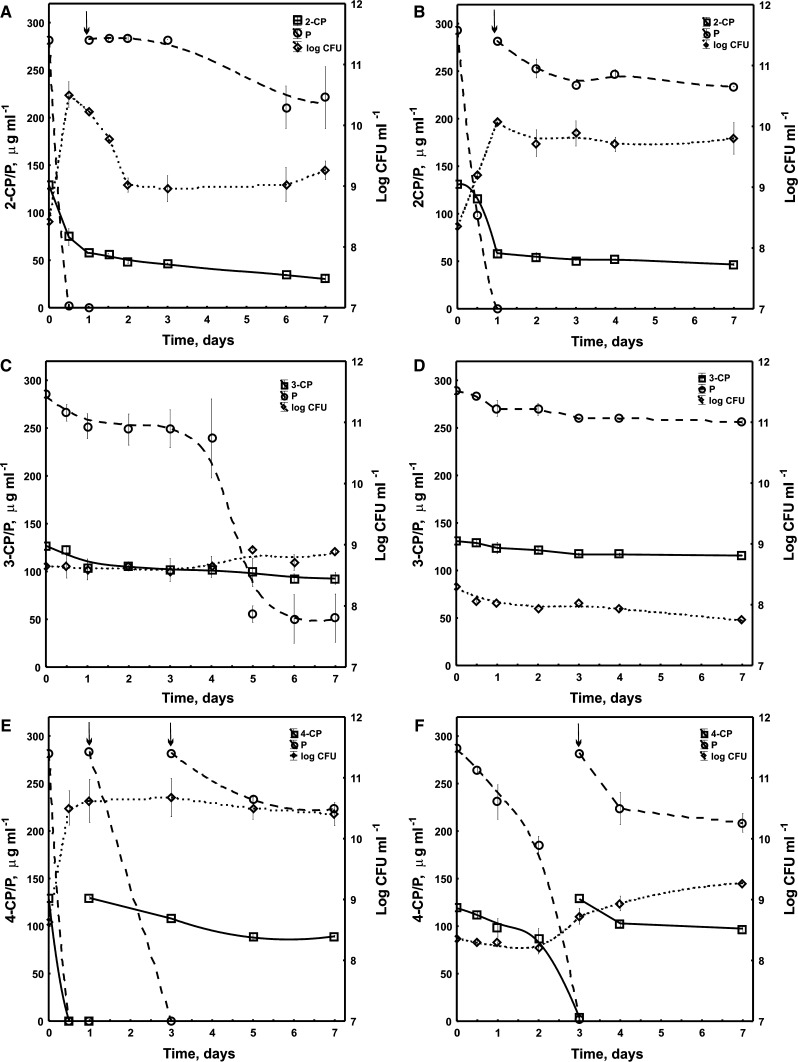
Co-metabolic degradation of 2-CP, 3-CP and 4-CP in the presence of P by *S. maltophilia* KB2 and survival of bacteria. **a**, **c, e**—exposed cells; **b**, **d**, **f**—unexposed cells. Each *symbol* represents the means of three replicates. *Arrows* indicate introduction of substrate(s) into the culture

The co-metabolic degradation study indicated significant differences in the course of degradation of each monochlorophenol by exposed and unexposed cells. The exposed cells degraded completely the first dose of 4-CP within 12 h. During the next 6 days they removed only 30 % of the second dose. Simultaneously, in the presence of 4-CP *S. maltophilia* KB2 degraded completely two doses of P. The first one it utilized within 12 h and the second one within 2 days. The third dose of P bacteria metabolized in 20 % during the next 4 days (Fig. 2e). Contrary to 4-CP, 2-CP and 3-CP bacteria degraded partially. During the first 7 days KB2 transformed 75 and 25 % of 2-CP and 3-CP, respectively (Fig. 2a, c). Data obtained from a parallel study on the co-metabolic degradation of monochlorophenols by unexposed *S. maltophilia* KB2 indicated the lower degradative potential of such bacteria towards monochlorophenols in comparison with the exposed ones. The unexposed bacteria completely degraded the first dose of 4-CP and P within 3 days (Fig. 2f), while during the next 4 days they transformed only 20 and 25 % of the second dose of 4-CP and P, respectively. In comparison, the unexposed bacteria transformed 65 % of the initial dose of 2-CP in the presence of P during 7 days, however they were unable to transform 3-CP (Fig. 2b, d). The highest bacterial counts were estimated in the medium with exposed cells in the presence of 4-CP and P (Fig. 2e), while in the presence of 3-CP and P the number of cells was the smallest (Fig. 2c, d).

### Activity of catechol 2,3-dioxygenase

As shown in Table 1, the relative activity of catechol 2,3-dioxygenase in bacteria culturing with 4-CP and P reached 81.74 % of its activity in phenol-degrading cells. However, in bacteria co-metabolized 2-CP and P it was significantly lower (22.87 %). In turn, the activity of 2,3-dioxygenase in the cells cultured with 3-CP and P was completely inhibited.

**Table 1:** Relative activity of catechol 2,3-dioxygenase of *S. maltophilia* KB2 during transformation of 2-CP, 3-CP and 4-CP in the presence of P

Substrate(s)	Relative activity (%)
P	100.00 ± 18.72 a
P + 2-CP	22.87 ± 0.63 b
P + 3-CP	0.00 ± 0.00 c
P + 4-CP	81.74 ± 2.41 d

The data presented are means of three replicates. The plus/minus values represent standard deviation. The different letters indicate significant differences (*p* < 0.05, LSD test), considering the effects of monochlorophenols on enzymes activity

### FAMEs analysis

**Fig. 3:**
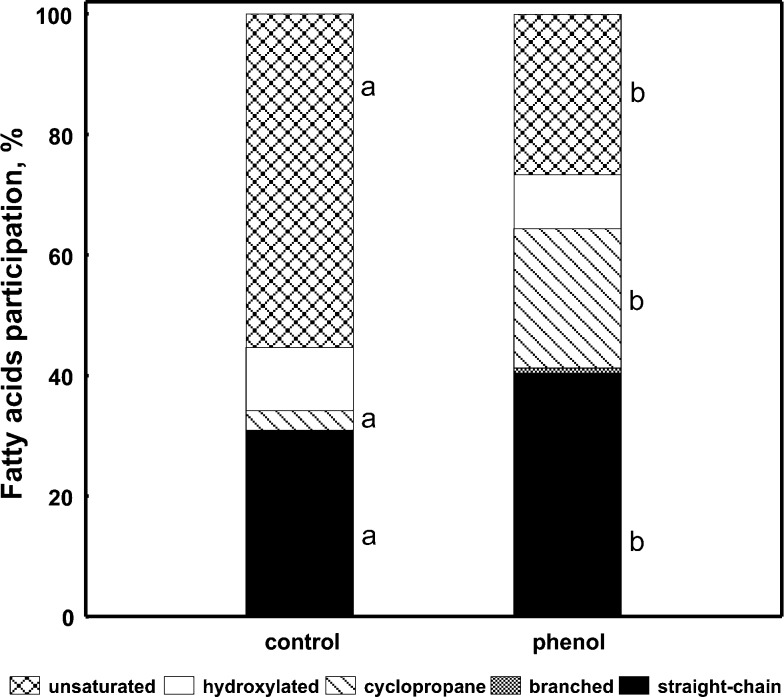
Proportions of different groups of fatty acids in phenol-degrading *S. maltophilia* KB2 and control cells. Data are representative of three individual experiments. The *different letters* indicate significant differences (*p* < 0.05, LSD test) considering the effects of the medium

At first, to illustrate FAME variability in phenol-degrading *S. maltophilia* KB2 during degradation of P, the whole-cell derived fatty acids were directly extracted from these bacteria and control cells. The detailed analysis of FAME profiles indicated significant increase in the content of cyclopropane and straight-chain fatty acids, with a simultaneous decrease of the unsaturated fatty acids in phenol-degrading cells as compared to the control cells (Fig. 3).

**Fig. 4:**
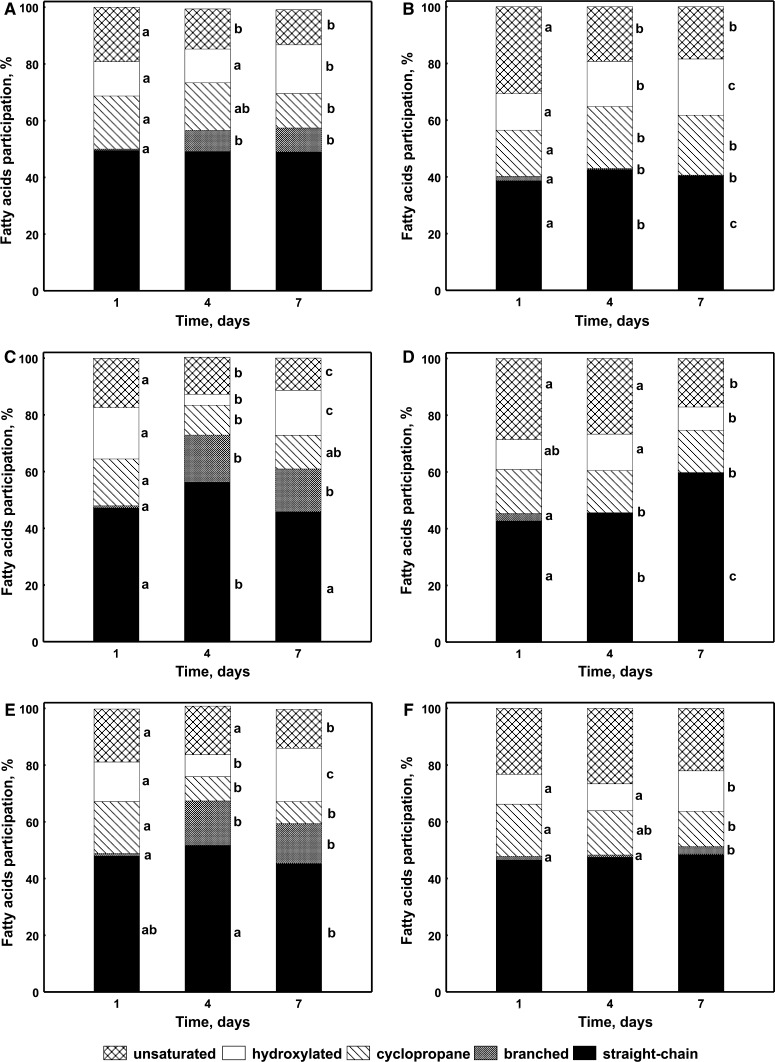
Proportions of different groups of fatty acids in *S. maltophilia* KB2 growing in the presence of 2-CP and P (**a**, **b**), 3-CP and P (**c**, **d**), 4-CP and P (**e**, **f**) on days 1, 4 and 7. **a**, **c**, **e**—exposed cells; **b**, **d**, **f**—unexposed cells. The *different letters* indicate significant differences (*p* < 0.05, LSD test) considering the effects of the incubation period

**Fig. 5:**
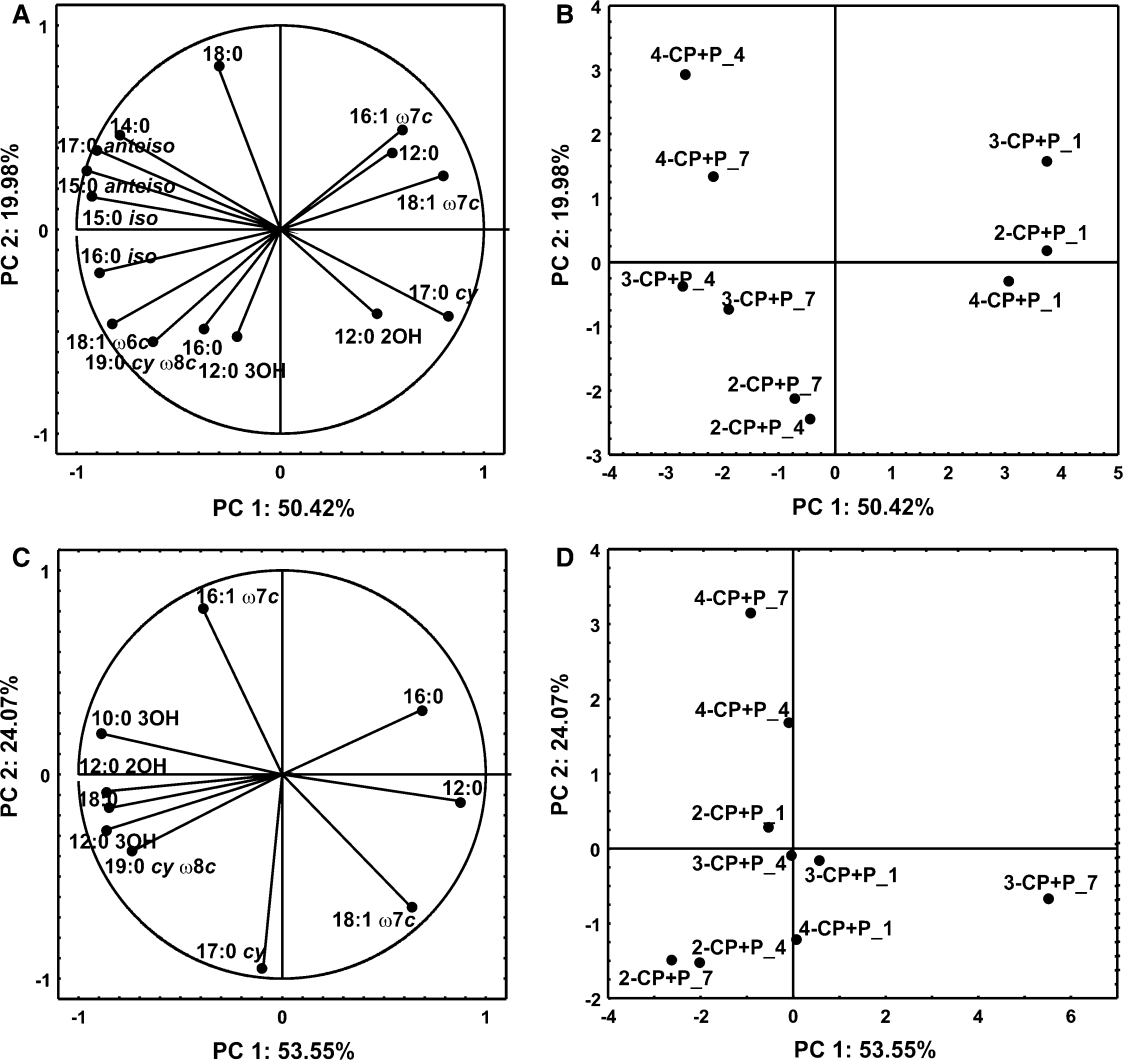
PCA of FAMEs during co-metabolic transformation of monochlorophenols in the presence of P by *S. maltophilia* KB2. **a**, **b**—exposed cells; **c**, **d**—unexposed cells. **a**, **c**—correlation of fatty acids with PC1 and PC2; **b**, **d**—projection of FAMEs on the plane defined by PC1 and PC2. 2-CP + P_X—FAMEs isolated from bacteria degrading 2-CP and P; 3-CP + P_X—FAMEs isolated from bacteria degrading 3-CP and P; 4-CP + P_X—FAMEs isolated from bacteria degrading 4-CP and P; X—sampling day

The analysis of FAMEs isolated from *S. maltophilia* KB2 exposed to P during the co-metabolic degradation of monochlorophenols indicated that both the earlier exposure of bacteria to P and the time of incubation affected their fatty acid composition. In bacteria exposed to P, regardless of the isomer added, a remarkable increase in the branched fatty acid content on day 4 was observed. On the last sampling day the increase in the percentage of hydroxylated fatty acids in these bacteria was also significant. The increase in the content of branched and hydroxylated fatty acids was correlated with the reduction in the contribution of cyclopropane and unsaturated fatty acids (Fig. 4a, c, e). The PCA analysis indicated that all FAMEs extracted from bacteria on day 1 were distinct in comparison with the other fatty acids along the first axis, and they were mainly characterized by higher content of 16:1 ω7*c,* 18:1 ω7*c* and 17:0 *cy* (Fig. 5a, b). Furthermore, the decrease in the percentage of these fatty acids in FAMEs profiles obtained for bacteria on days 4 and 7 was correlated with a simultaneous increase in the content of branched fatty acids 15:0 *iso,* 15:0 *anteiso,* 16:0 *iso,* 17:0 *anteiso*, and hydroxylated fatty acid 12:0 3OH (Fig. 5a, b).

Contrary to the presented results, in the cells unexposed to P the increase in the degree of membrane saturation was connected with different classes of fatty acids and depended on the type of isomer used. In the presence of 2-CP the increase in the content of hydroxylated (10:0 3OH, 12:0 2OH, 12:0 3OH) and cyclopropane (17:0 *cy*) fatty acids on day 4 was recorded (Figs. 4b and 5c, d). At the same time, in the presence of 3-CP a remarkable increase in the percentage of straight-chain (12:0, 16:0) fatty acids in the FAME pattern was revealed (Fig. 4d and 5c, d). In turn, the presence of 4-CP in culture medium caused a remarkable increase in the abundance of hydroxylated fatty acids (10:0 3OH, 12:0 2OH, 12:0 3OH) (Figs. 4f and 5c, d).

## Discussion

The strain *Stenotrophomonas maltophilia* KB2 was described previously as a good degrader of different monocyclic aromatic hydrocarbons, such as phenol, benzoic acid and its derivatives. However, it was not able to degrade monochlorophenols as a sole carbon source (Guzik et al. 2009). Because co-metabolic degradation of monochlorophenols by KB2 has not been studied extensively, the aim of this research was to investigate this phenomenon as well as to find out and fully explain adaptive mechanisms of bacteria involved the alterations in their cellular fatty acid composition under co-metabolic conditions.

The results of this study indicated that *S. maltophilia* KB2 was capable of completely degrading 4-CP in the presence of P, while the degradation of 2-CP and 3-CP was incomplete. It was also confirmed that the efficiency of the co-metabolic degradation of monochlorophenols by these bacteria depended on the earlier exposure of bacteria to phenol and the position of the chlorine substituent in the aromatic ring. The exposure of bacteria to the growth substrate allowed them to induce enzymes responsible for the degradation of phenol and in a consequence increased the rate of chlorophenols utilization. It was evidenced that the exposure of bacteria to easily degradable aromatic compounds can accelerate the removal of other hard-to-degrade compounds and reduce the time needed for their biodegradation. In another study (Wojcieszyńska et al. 2013) reported that *S. maltophilia* KB2 was able to degrade 90 % of 2-CP added to the medium at a concentration of 65 mg l^−1^ in the presence of phenol at a concentration of 375 mg l^−1^ during one week of incubation. Contrary to our studies, they followed the breakdown of 2-CP in the presence of the growth substrate, which was added every 24 h to the initial level. During this experiment bacteria degraded in total 59 mg l^−1^ of 2-CP, while in our experiment at the same time they were able to degrade 98 mg l^−1^ of this compound.

Differences in the activity of catechol 2,3-dioxygenase, the main enzyme induced by phenol as the growth substrate, in the presence of different isomers of monochlorophenols indicated the diverse sensitivity of this enzyme depending on the position of the chlorine substituent in the aromatic ring. The lack of dioxygenase activity in the presence of 3-CP was in an agreement with the negligible co-metabolic degradation of this isomer, while the inhibition of this enzyme about 20 % in comparison with its activity in phenol-degrading bacteria did not affect the degradation of 4-CP. The competitive inhibition of catechol 2,3-dioxygenase by 2-CP and 4-CP was also confirmed by Wojcieszyńska et al. (2013). Similarly, the ability of the *Alcaligenes* sp. A7-2 strain to degrade phenols in order P > 4-CP > 2-CP > 3-CP was observed by Menke and Rehm (1992). They connected this order with the configuration of hydroxyl and the chloride substituent in the aromatic ring and the facility of the introduction of the second hydroxyl group into the substrate. Because of suicide inactivation of catechol 2,3-dioxygenase by 3-halocatechols, 1,2-catechol dioxygenase is more often involved in the ring cleavage of aromatics with one or two chlorine substituents (Arora and Bae 2014). In the cells of *Stenotrophomonas maltophilia* KB2 growing in the presence of phenol the activity of catechol 1,2-dioxygenase was found to be negligible (Guzik et al. 2009).

It has been demonstrated that the composition of fatty acids extracted from *S. maltophilia* KB2 differed depending on the degraded compounds, prior exposure to phenol, and the time of incubation. During the degradation of phenol as a single carbon source bacteria regulated the fluidity of their membranes by the increase in the contents of cyclopropane (17:0 *cy*, 19:0 *cy* ω8*c*) and straight-chain (12:0, 16:0) fatty acids. The increased abundance of these fatty acids made the membrane less permeable because cyclopropane fatty acids caused a similar effect as increased bilayer thickness, while straight-chain fatty acids made it more rigid through strict packing of their acyl chains (Chen et al. 1995; Grogan and Cronan 1997). It should be noted that the most crucial alterations in fatty acid composition were correlated with the highest activity of catechol 2,3-dioxygenase.

The earlier exposure of *S. maltophilia* KB2 to phenol had a different influence on the composition of cellular fatty acids during the co-metabolic degradation of monochlorophenols as compared to unexposed cells. The main difference between these cells was connected with the increase in the abundance of branched fatty acids in the exposed cells. However, this mechanism was not confirmed in the unexposed cells. As Lindström et al. (2006) reported, the incorporation of branched fatty acids in phospholipids stabilizes the gel phase of the membrane bilayers and makes them more rigid. It should be pointed out that branched fatty acids are *de novo* synthesized, and therefore the described changes in the membrane structure are possible only in growing cells (Fujita et al. 2007). For this reason, their content may be considered as an indicator of the degradation of monochlorophenols by *S. maltophilia* KB2 exposed to phenol. The branched fatty acids are not typical for Gram-negative bacteria. However, their appearance in FAMEs profiles of Gram-negative bacteria under chemical stress in some reports was confirmed (Haack et al. 1994; Mrozik et al. 2005; Nowak and Mrozik 2016). Furthermore, the variability of the cellular fatty acids in dependence to the co-metabolized isomer of monochlorophenols was also connected with the earlier exposure of bacteria to phenol. In the exposed cells this relation was not distinct, while in unexposed ones the most toxic and the most barely degradable 3-CP affected the decrease of membrane permeability through the increased content of straight-chain fatty acids. In turn, in the presence of 2-CP the same effect was achieved by the increase in the content of cyclopropane fatty acids. This dependence was not observed during the co-metabolic degradation of monochlorophenols by *Pseudomonas* sp. CF600 (Nowak and Mrozik 2016).

In conclusion, the earlier exposure of *S. maltophilia* KB2 to phenol can accelerate the co-metabolic biodegradation of barely degradable chlorophenols through the induction of required enzymes and correspondent changes in their fatty acid composition. In particular, the changes in the contribution of branched fatty acids in FAME profiles may be proposed as a good indicator of the presence of chlorophenols in the batch cultures. However, further studies are needed to evidence a potential application of this strain in bioremediation of soil and water environments contaminated with chlorophenolic compounds.
